# Swallowing interventions for older in-hospital patients: have we appropriately selected the desired outcomes?

**DOI:** 10.1590/1806-9282.20231403

**Published:** 2024-05-03

**Affiliations:** 1 Hospital Moinhos de Vento, Internal Medicine Service – Porto Alegre (RS), Brazil.; 2 Hospital Universitário de Canoas – Canoas (RS), Brazil.

Pneumonia is a common condition that leads to hospitalization among older adults, with six times higher incidence of hospital admissions for individuals over 80 years of age versus the younger individuals^1^. As microaspiration is the primary pathogenic mechanism for most cases of pneumonia, aspiration pneumonia is better understood as a continuum that encompasses both community- and hospital-acquired pneumonia^1^. The lung microbiome is maintained through a delicate balance of bacterial migration from the oropharynx to the lungs, primarily through microaspiration, and elimination via ciliary clearance and coughing^1,2^. When this balance is disrupted by bacterial and viral virulence^1,2^, or through macroaspiration, then infection, inflammation, and tissue damage result. Given the importance of aspiration in the pathophysiology of all pneumonia and the significant number of older adult patients hospitalized with pneumonia who have impaired swallowing, with studies indicating that nearly 92% of this population exhibit oropharyngeal dysphagia^1^, it is critical to understand the preventive measures to reduce the risk of recurrence or to improve outcomes of hospitalized patients with pneumonia, such as the involvement of Speech Language Pathologists with a rehabilitation program, changing the viscosity of liquids, and the consideration of tube feeding^1,3^. Screening and specific measures aimed at preventing aspiration are recommended by international organizations for hospital quality and safety^4^. However, generally enteral feeding through nasogastric tube placement did not lead to a reduction in pneumonia risk^1^. Postpyloric or gastrostomy feeding methods are not superior to the nasogastric tube^1^. In a Cochrane meta-analysis, the use of percutaneous endoscopic gastrostomy compared to nasogastric tube feeding showed no significant difference in pneumonia risk (RR 0.7 [95%CI 0.46–1.06]) or mortality risk (RR 0.86 [95%CI 0.58–1.28]), regardless of the follow-up duration (evidence quality rated as low and very low, respectively)^5^.

With respect to swallowing interventions with Speech Language Pathologists, the potential benefits can be significant^3^, including a reduction in the length of hospital stay, involving compensatory techniques and exercises for muscle rehabilitation, thus improving food and fluid intake, preserving hydration and nutrition, and minimizing the risk of macroaspiration^3,6^. However, when we evaluate recurrence of pneumonia and mortality with behavioral swallowing interventions alone, we did not find significant differences in pneumonia risk (OR 0.56 [95%CI 0.31–1.0]), and for mortality outcomes, the results did not show any improvement with any type of swallowing interventions (OR 1 [95%CI 0.66–1.52]), with evidence quality rated as very low and moderate, respectively^7^.

Similarly, the use of thickened liquids did not reduce mortality or pneumonia risk, but studies suggesting this may impact adherence and a tendency toward an increased risk of dehydration and weight loss^8^. Although there is an improvement in the physiology of swallowing with thick fluids, this is not necessarily linked to a reduction in respiratory complications, considering that the risk of developing aspiration pneumonia is possibly reduced by the aspiration of pure and thin water than by the aspiration of thick fluids because aquaporins allow the removal of water from the air spaces after laryngotracheal aspiration, reducing the risk of aspiration pneumonia if aspiration occurs^6^.

These findings raise a crucial question regarding the selection of outcomes in clinical trials involving older adult patients: Are we appropriately choosing the outcomes to evaluate? These results suggest a trend toward unmodifiable risk factors among the oldest individuals with oropharyngeal dysphagia, highlighting the importance of gaining a better understanding of our current position and desired goals^9^. For instance, none of the clinical trials included in the Cochrane meta-analysis on swallowing therapist interventions assessed quality of life outcomes, which are highly relevant patient-centered outcomes for frail and older adults^3,7^. In the meta-analysis focusing on thickened liquids, worse quality of life scores were reported in the intervention group^8^. However, when evaluating protocols involving the provision of free water for oropharyngeal dysphagia, taking into account aspects such as oral hygiene, time of ingestion, type of liquid, and cognitive characteristics for ingestion safety^6,10^, studies have shown that patients’ perceptions of swallow-related quality of life improve (as assessed by standardized questionnaires like SWAL-QOL), without an increase in pulmonary complications^6,10^.

Finally, it is crucial to comprehend the implications of these interventions particularly in middle- and low-income healthcare settings. Implementation of such interventions may potentially increase concerns among families during the discharge process, raising the length of hospital stay, thereby complicating matters. Consequently, there is an imperative for enhanced outcome selection in clinical trials aimed at interventions for dysphagia in hospitalized older patients. It is crucial that these trials prioritize patient-centered measures, including quality of life, length of hospital stays, and the duration of post-hospitalization survival. Additionally, it is essential to consider the viewpoints of family members and caregivers regarding these outcomes.

Recognizing the significance of a “core outcome set” is paramount in assessing the efficacy and impact of such interventions. This core setting, comprising a standardized selection of key outcomes, facilitates comparability and enables researchers to draw meaningful conclusions about the effectiveness of dysphagia interventions. By utilizing a core outcome set, we can ensure that future research in this field is robust, patient-focused, and capable of producing insights that genuinely benefit both patients and their caregivers. The emphasis should not solely be on pneumonia recurrence or mortality rates among older patients with multiple risk factors and frailty. We are certainly striving to do the right thing, but we may have chosen the wrong path.
